# Clinical medication reviews in elderly patients with polypharmacy: a cross-sectional study on drug-related problems in the Netherlands

**DOI:** 10.1007/s11096-015-0199-8

**Published:** 2015-11-23

**Authors:** Sek Hung Chau, Aaltje P. D. Jansen, Peter M. van de Ven, Petra Hoogland, Petra J. M. Elders, Jacqueline G. Hugtenburg

**Affiliations:** 1 Department of Clinical Pharmacology and Pharmacy, VU University Medical Center, De Boelelaan 1117, 1081 HV Amsterdam, Netherlands; 2 The EMGO Institute for Health and Care Research, VU University Medical Center, Amsterdam, Netherlands; 3 Department of General Practice and Elderly Care Medicine, VU University Medical Center, Amsterdam, Netherlands; 4 Department of Epidemiology and Biostatistics, VU University Medical Center, Amsterdam, Netherlands; 5 Department of Pharmaceutical Affairs, Service Apotheek Beheer B.V., Enter, Netherlands

**Keywords:** Clinical medication reviews, Community pharmacists, Drug-related problems, Netherlands, Older adults, Patient care, Pharmaceutical care

## Abstract

*Background* Knowledge of drug-related problems (DRPs) identified in the medication of home-dwelling elderly patients with polypharmacy has been based predominantly on medication reviews conducted in research settings rather than in daily practice. *Objective* To evaluate the prevalence of DRPs identified by means of a clinical medication review (CMR) and the implementation rate of proposed interventions in a large group of older patients with polypharmacy in the daily practice of community pharmacies. *Setting* 318 Dutch community pharmacies. *Method* A cross-sectional study based on CMR-data of 3807 older patients (≥65 years) with polypharmacy (≥5 drugs) completed between January and August 2012. Data were extracted from community pharmacists’ databases and entailed: year of birth, gender, dispensing data, number and nature of identified DRPs, consultations performed, proposed and implemented interventions. *Main outcome measure* Prevalence of DRPs, drug classes involved in overtreatment and undertreatment, and proposed and implemented interventions. *Results* A median of two DRPs (interquartile range 1–4; mean 3.0) was identified per patient. The DRP-categories overtreatment (25.5 %) and undertreatment (15.9 %) were found most frequently. 46.2 % of the proposed interventions to solve DRPs were implemented as proposed, in 22.4 % of cases, the intervention differed from the proposal. In 31.3 % of cases no intervention was implemented. *Conclusion* By conducting a CMR community pharmacists identified a median of two DRPs in older patients with polypharmacy. Overtreatment and undertreatment accounted for 41.4 % of the DRPs identified. In dealing with DRPs, pharmacists proposed a variety of interventions of which the majority (69.9 %) was either implemented or led to alternative interventions. A set of explicit criteria should be applied during a CMR to solve and prevent DRPs.

## Impact of findings on practice

- Clinical medication reviews routinely conducted by community pharmacists contribute to a reduction of DRPs in the medication of older patients with polypharmacy.
- The application of explicit criteria (STOPP/START or the Amsterdam Tool) in a clinical medication review may be a useful method to identify (potential) DRPs frequently found in the medication of older patients with polypharmacy.

## Introduction

Conducting medication reviews is a method often recommended to identify and solve drug-related problems (DRPs) in order to optimise drug treatment and to improve patient health outcomes [1–3]. DRPs can be defined as events involving drug treatment that are actually or potentially harmful to a patient’s health or prevent patients to optimally benefit from treatment [4]. The term medication review is used for a broad array of interventions, which are aimed to identify and solve DRPs. A clinical medication review (CMR) entails a review involving communication with the patient while considering treatment in the context of the patient’s underlying condition and symptoms [2]. The advantage of patient involvement is the identification of more DRPs and the possibility to identify DRPs which are particularly relevant to the patient and therefore should be tackled with priority [5–8]. The risk of DRPs increases with age, number of diseases and number of drugs prescribed [9, 10]. Older patients with polypharmacy are most likely to benefit from a medication review [11, 12] as they are more vulnerable to complications and admission to hospital caused by DRPs [9]. Knowledge of DRPs identified in home-dwelling older patients with polypharmacy and their origin predominantly has been derived from medication reviews in research settings rather than in daily practice of community pharmacy. Medication reviews conducted in research settings identified 2.5–10 DRPs in older patients with polypharmacy [7, 13, 14]. Differences in the number of identified DRPs can be explained by variations in the target population, experience of the pharmacist conducting the review, extent of patient involvement, access to General Practitioner’s (GP) medical records, and patient-related factors such as age, number of diseases, number of drugs, and living conditions (e.g. residential versus home dwelling) [15–17].

Knowledge of the type of DRPs in the elderly identified by means of a CMR has been mainly obtained from studies of 100 up to 400 patients. DRPs most frequently identified relate to drug selection problems (27.8–42.0 %) [7, 13, 14], over- and underdosing (10.8–30.0 %), and over- (9.0–29 %) and undertreatment (3.0–27.9 %) [7, 13, 14, 18, 19]. However, data on DRPs identified by means of a CMR in daily practice is scarce. A study on Australian CMRs conducted by accredited community pharmacists nationwide between 1998 and 2005 in daily practice revealed on mean 4.6 DRPs per patient. DRPs most frequently identified were drug selection problems (24.9 %), toxicity, adverse reactions and side effects (19.7 %), and undertreatment (15.7 %). Over- and underdosing comprised 8.9 % of the DRPs [17].

A limited number of studies has reported whether and how proposals for interventions forthcoming from CMRs were dealt with, using heterogeneous outcome measures [14, 19–24]. Agreement of the prescriber with the proposal was reported in two studies and amounted to 41.8 and 75.3 % respectively [19, 20]. Changes observed in clinical or pharmacy records on the basis of CMRs were reported as implementation rates between 17 and 85 % [14, 20–23]. However, as yet data on implementation rates in daily practice are lacking.

In the Netherlands, community pharmacists started to conduct CMRs in older patients with polypharmacy on a large scale as part of the care contractually agreed with health insurers. This enabled the implementation of proposed interventions in daily practice and the current study on DRPs. Standard health insurance is legally compulsory for every person living or working in the Netherlands [25]. Dutch community pharmacies use computerised systems to register dispensed medication which also enables registration of over-the-counter medication [26]. Most patients are registered in a single pharmacy while shopping behaviour (i.e. visiting more than one pharmacy) tends to be low [27]. When a patient visits another pharmacy and patient permission is obtained, the patients’ main pharmacy is informed about the dispensed medication and dosing advices, electronically by using a secured connection or by a fax message. Prescribers are also obliged to list the indication when prescribing a drug that appears on a list of 23 selected drugs with a small therapeutic window, and to actively inform pharmacists in the case of an abnormal renal function [28]. This enables community pharmacists to keep medication records complete and detect drug–drug interactions among others. These Dutch requirements are quite unique in comparison with pharmacy practice in other countries [27].

## Aim of the study

The present study aimed to investigate the number of DRPs in the elderly with polypharmacy identified by means of a CMR in daily practice of community pharmacy and the implementation rate of the proposed interventions.

## Ethical approval

Since anonymised data extracted from the community pharmacists’ databases were used, ethical approval was not required according to Dutch legislation.

## Method

### Design and setting

A cross-sectional study was carried out based on CMRs in older patients with polypharmacy completed between January and August 2012. Data were acquired from all 318 Dutch community pharmacies affiliated to Nederlandse Service Apotheek Beheer (16 % of all community pharmacies), a franchise organisation of independent community pharmacies, located across the Netherlands. Patient characteristics, prevalence of identified DRPs, and proposed and implemented interventions have been registered in a database using the Service Apotheek Medication Review Tool (SAMRT) in NControl, a collection of web applications supporting the process of pharmaceutical care.

### Study population

In 2012 eight Dutch health insurance companies (combined market share >85 %) contracted CMRs to be conducted by community pharmacists though imposing different patient criteria for reimbursement. For example, one insurer excluded patients with multidose drug dispensing systems (MDD) from CMR. In MDD, medication are packaged and ordered in separate compartments for each dose occasion and labelled with patient data, date and time of intended intake and drug contents. Other insurers only reimbursed CMRs for patients using specific drug classes such as drugs for COPD or specific combinations of cardiovascular drugs.

However, the CMRs of all patients could be registered in the SAMRT database. From this database, the study population for the current study was composed, consisting of patients aged 65 years or older with polypharmacy. Polypharmacy is defined as the use of at least five drugs indicated for the treatment of a chronic disease at the anatomical therapeutic chemical (ATC)-5 level in the past four months. In case of preparations for sensory organs, only drugs intended for long-term use were included (ATC-class S01E,F,G). Anti-infectives (ATC-class J, G01, S01A,C, S02A,C) are intended for short-term use and therefore have been excluded. Drugs less likely to cause relevant DRPs, such as dermatologicals (ATC-class D) and topical products (ATC-class M02), were also excluded for the calculation of the total number of drugs.

### Clinical medication review

CMRs were conducted by community pharmacists who completed a medication review training accredited by the Royal Dutch Pharmacists Association. Pharmacists followed a method that evolved into the STRIP method [29]. Each CMR started with a semi-structured interview with the patient. In this interview, the aim and use of the patients’ medication were discussed as well as any DRP experienced or perceived by the patient. In conducting a CMR pharmacists were supported by the SAMRT. Different pharmacists may have been involved in conducting the CMRs, if multiple pharmacists were employed in a pharmacy. The medication review tool guides the pharmacist through the medication history of the patient to identify possible DRPs. Proposed interventions were discussed with the prescriber, unless the intervention was not regarding a change of medication i.e. repeating an inhaler instruction or creating a dosing schedule. Final proposals to be implemented were discussed with the patient by the pharmacist. Furthermore, SAMRT served as a registration tool to CMR proceedings including a summary of the patient interview, registration of diseases (International Classification for Primary Care, ICPC code), DRPs identified, whether the prescriber and/or patient was consulted to solve the DRP and proposed and implemented interventions.

### Data collection

Data of patients of 65 years and older with polypharmacy were extracted from the community pharmacists’ databases (pharmacy information and administration system and SAMRT). Data entailed: year of birth, gender, dispensing data, number and nature of identified DRPs, consultations performed, proposed interventions and those implemented.

### Outcome measures and data analysis

Descriptive analyses were performed on anonymised data of the outcome measures using statistical software (SPSS version 20.0). Characteristics have been presented as number and mean [with standard deviation (SD)]. For variables that were not normally distributed, both the median [with interquartile range (IQR)] and mean is reported in order to be able to compare results with the existing literature. Percentages of drug classes involved in the two largest DRP categories were calculated by dividing the number of DRPs involving the drug class on the total number of DRPs in that category.

## Results

### Patient characteristics

3807 CMRs were conducted in older patients with polypharmacy across 258 of 318 pharmacies. The participating older adults had a mean age of 78 years; 57.9 % were female. Patients used a median of nine drugs (IQR 7–11; mean 9.54) for the treatment of a chronic disease (see Table 1). No patient received more than one CMR.

**Table 1:** Patient demographics

Characteristics	Total population
	n = 3807
*Gender*	
Male	42.1 %
*Age (years)*	
Mean (SD)	78 (7.68)
Range	65–102
*Number of chronically used drugs*	
Median (IQR)	9 (7–11)
Mean (SD)	9.54 (3.40)
Range	5–27
*Number of DRPs*	
Median (IQR)	2 (1–4)
Mean (SD)	3.00 (2.26)
Range	1–26

*SD* standard deviation, *IQR* interquartile range

### Number of DRPs and DRP prevalence

Table 2 lists the number of the various DRPs that were identified. In total, 11,419 DRPs were found in 3807 patients. A median of two (IQR 1–4) DRPs was found per patient (mean: 3.0). Overtreatment (25.5 %) and undertreatment (15.9 %) were the most common DRPs. Other DRP-categories each contributed 5.0–8.5 % to the total number of DRPs reported. Inappropriate dosage form was the least common DRP (0.8 %).

**Table 2:** Number of DRPs per category

DRP categories	n	%
Overtreatment	2915	25.5
Undertreatment	1814	15.9
Drug not effective	975	8.5
Contra-indication	971	8.5
Side effect	923	8.1
Difficulty using dosage form	756	6.6
Interaction	664	5.8
Non adherence	645	5.6
Dose too low	622	5.4
Dose too high	568	5.0
Inappropriate dosage form	96	0.8
Miscellaneous problem^a^	470	4.1
Total DRPs	11,419	100

^a^Besides drug-related problems, the category ‘miscellaneous problem’ also contained non-drug-related problems, for example, lifestyle advice given such as smoking cessation

### Drug classes involved in overtreatment and undertreatment

Drug classes involved in the DRP categories overtreatment and undertreatment have been listed in Table 3. Drug classes most frequently involved in overtreatment are drugs for peptic ulcer and gastro-oesophageal reflux disease (GORD, 10.2 %), antithrombotic agents (6.7 %) and lipid modifying agents (5.2 %).

**Table 3:** Drug classes involved in DRP categories overtreatment and undertreatment

		n	Percentage (%)
*Drug classes involved in overtreatment*			
A02B	Drugs for peptic ulcer and GORD	296	10.2
B01A	Antithrombotic agents	195	6.7
C10A	Lipid modifying agents	153	5.2
A06A	Drugs for constipation	103	3.5
N05B	Anxiolytics	83	2.8
N05C	Hypnotics and sedatives	75	2.6
N06A	Antidepressants	71	2.4
C03C	High-ceiling diuretics	66	2.3
C07A	Beta blocking agents	60	2.1
N02B	Other analgesics and antipyretics	60	2.1
*Drug classes involved in undertreatment*			
C10A	Lipid modifying agents	53	2.9
B01A	Antithrombotic agents	48	2.6
A11C	Vitamin A and/or D	46	2.5
A12A	Calcium	26	1.4
A10B	Blood glucose lowering drugs, excl. insulin	23	1.3
A02B	Drugs for peptic ulcer and GORD	17	0.9
C01D	Vasodilators used in cardiac diseases	15	0.8
M05B	Drugs affecting bone structure and mineralisation	15	0.8
C09A	ACE inhibitors	14	0.8
C07A	Beta blocking agents	11	0.6

Percentage given is the percentage of a drug class reported in the DRP category. *GORD* gastro-oesophageal reflux disease

The majority of the drugs for peptic ulcer and GORD involved in overtreatment were proton-pump inhibitors [PPI, (n = 281, 94.9 %)]. PPIs were classified as overtreatment because there was no clear indication or gastroprotective medication was no longer needed since a non-steroidal anti-inflammatory drug (incl. acetylsalicylic acid) or selective serotonin reuptake inhibitor was stopped previously. Vitamin K antagonists (n = 47) were regarded as overtreatment because the indication was unclear or unknown to the patient and pharmacist. However, after consultation with the prescriber, these drugs frequently turned out to be indicated. In most cases clopidogrel was registered as overtreatment (n = 38), mainly because the patient had used it longer than 1 year and treatment could be discontinued according to the current medical guidelines. Rosuvastatin contributed 50.3 % (n = 77) to the group of lipid modifying agents. Lipid modifying agents and antithrombotic agents were also involved in undertreatment, 2.9 and 2.6 % respectively, followed by vitamin A and/or D (2.5 %).

### Interventions

Proposed interventions have been listed in Table 4. Stopping to use a drug was suggested most frequently (19.6 %), second most suggested was monitoring of the patient (18.4 %), e.g. measuring of the blood pressure or performing a blood test. 46.2 % of the interventions were implemented as proposed. In 22.4 % of the cases, the intervention effectuated differed from the proposal and in 31.3 % of the cases, no intervention was performed. Reasons for not implementing an intervention included a rejection by the prescriber (27.5 %) or patient (11.9 %); and correction of the DRP in the time between the patient interview and the consultation with the physician (mostly a GP) or patient (13.1 %). Some interventions were not implemented immediately, but instead the patient was monitored to decide whether the intervention was indicated at a later moment (26.2 %). An overview of all interventions effectuated can be found in Table 5.

**Table 4:** Prevalence and type of proposed interventions after identification of the DRP by pharmacists

	Number	Percentage (%)	Implemented (%)	Other intervention (%)	No intervention (%)
Stop drug	2238	19.6	46.6	21.4	32.0
Provide monitoring	2099	18.4	52.8	23.1	24.1
Adjust dose	1684	14.7	43.3	25.1	31.6
Add drug	1601	14.0	36.3	27.4	36.3
Switch drug	1307	11.5	38.5	26.0	35.5
Provide education	1225	10.7	67.9	12.3	19.8
Synchronise medication	304	2.7	82.6	12.5	4.9
Switch dose form	176	1.5	60.2	24.4	15.4
Other	766	6.7	15.5	21.5	63.0
Total	11,419	100.0	46.2^a^	22.4^a^	31.3^a^

^a^These percentages were calculated based on the known outcomes (11,400) as a proportion of the total interventions (11,419). 0.2 % of the interventions (n = 19) was not attributed to a specific category

**Table 5:** Distribution of interventions implemented after identification of the DRP

	Percentage (%)
Information/advice given	17.3
Research conducted	14.6
Drug stopped	11.2
Dose changed/adjusted	7.9
Drug started	5.8
Drug replaced	5.5
Medication synchronised	2.4
Dosage form changed	1.1
Other/unspecified	4.2
No intervention	30.1
Total	100.0

## Discussion

The objective of this study was to investigate the number and nature of DRPs identified by means of a community pharmacist-conducted CMR in 3807 older patients with polypharmacy in daily practice. The implementation rate of interventions proposed to solve DRP was also investigated.

In older patients with polypharmacy whose medication was evaluated by means of a CMR three DRPs were identified on mean. This number is on the lower end of the range of 2.5–10 DRPs found for these patients in research settings [7, 13, 14] and comparable with the 4.6 DRPs previously found in daily practice [17]. However, comparison is problematic as DRPs are not normally distributed, but skewed to the right. Future studies should report median and IQR to allow better inter-study comparisons of the number of DRPs.

Overtreatment (25 %) was the most frequently identified DRP. The prevalence of overtreatment was within the range found in research settings (15.7–29 %) [7, 13, 14, 18]. Other studies also showed that cessation of medication was the most common recommendation following a CMR. In this respect, it should be noted that the past decade attention for the deprescribing of superfluous drug has increased [30]. Undertreatment (16 %) was the second most frequently identified DRP. This percentage is similar to that found in a previous study on CMRs in daily practice (15.7 %) [17] and also within the range obtained in research settings (3.0–27.9 %) [7, 13, 14, 18, 19]. Overtreatment and undertreatment accounted for 41.4 % of the DRPs identified. This implicates that explicit criteria developed for the detection of potentially inappropriate drugs and potentially omitted beneficial drugs, such as the STOPP/START criteria may be useful during a CMR as a tool to systematically screen the medication of the patient. However, rather than overtreatment and undertreatment, a larger part of DRPs concerned other DRPs (i.e. drug not effective, side effect, difficulty using dosage form). For additional support of the CMR process, explicit criteria addressing these DRPs may be useful, for example by using the recently developed Amsterdam Tool [31].

Drugs for peptic ulcer and GORD, antithrombotic agents and lipid modifying agents were most frequently involved in overtreatment. It is known that in some cases, drugs for peptic ulcer and GORD are used longer than indicated for GORD [32, 33] or gastroprotection. The unnecessary use of gastroprotective medication in some cases may be explained by the continuation of gastroprotective medication after the gastric problem enhancing drug was discontinued. Lipid modifying agents and antithrombotic agents were also frequently involved in overtreatment, as well as undertreatment. This could be explained by the fact that some patients with an indication are not treated, while others may receive a more potent drug than necessary (e.g. rosuvastatin while simvastatin has not been tried first). Drug classes used more often may have resulted in a higher percentage in a DRP-category. However, this still provides important information. By giving more attention to these drug classes, which caused a large number of DRPs, the number of DRPs may decrease.

Nearly half (46.2 %) of all pharmacist-proposed interventions were implemented as proposed. Furthermore, in an additional 23.7 % of cases, DRPs were dealt with in a manner different from the adjustment initially proposed. Apparently, discussion with GPs and patients in the CMR process influenced the choice of interventions and resulted in different adjustments. In research settings, implementation rates between 17 and 85 % were found [14, 19–24] and implementation rates were found to be generally higher when pharmacists had more clinical experience, had access to GP medical records and when a patient interview was part of the review process [34]. Higher implementation rates have also been found in studies focussing on selected DRPs [19] or in studies in which only a limited number of interventions were proposed [22]. Some of these factors may have had an effect on the implementation rate in the present study. The pharmacists in the present study proposed many interventions on a wide range of DRPs. This may have had a negative effect on the implementation rate whereas the patient interview may have had a positive effect. Although community pharmacists were specifically trained to conduct CMRs, it is likely that some community pharmacists still had limited experience in conducting CMRs, largely because in the Netherlands routinely conducted CMR were only reimbursed from 2012 on a larger scale. Finally, for effectively addressing DRPs, close cooperation with the GP seems important [34]. Unfortunately, there were no data providing information to what extent this cooperation was practised.

To appreciate the findings of the present study some points that may have affected the results must be addressed. First, the SAMRT has been designed to support pharmacists in conducting CMR in a structured manner on a routine basis, not as a registration system for research purposes. This might have resulted in a registration bias, due to variability in the registration of DRPs which in turn would have affected the findings on number and nature of DRPs and implementation rates. However, arguing against this potential bias as well as being a strength of this study, is that all pharmacists completed the same training and used the same SAMRT tool. Second, requirements for CMR reimbursement of pharmacists by the health insurers involved led to some heterogeneity in the patient population. Regarding the exclusion of patients using a MDD: this might have led to a lower number of DRPs, because patients using MDD are known to have more DRPs [35]. Some insurers only reimburse CMRs for patients using specific drug classes such as drugs for COPD or specific combinations of cardiovascular drugs, which might have influenced the number of DRPs. Pharmacists may also have selected those patients who they assumed to benefit the most, i.e. are more likely to suffer from DRPs, which might have increased the number of DRPs. On the other hand, other pharmacists may have approached all eligible patients until they met the number of CMRs they contractually agreed to conduct, which might have influenced the number of DRPs as the selection was more random, i.e. also patients with less DRPs. To what extent these issues affected the findings overall, cannot be determined. Third, the experience of the pharmacists has not been taken into account. Although all pharmacists were trained to conduct CMRs in the same fashion, this may have influenced the number of DRPs identified and the percentage of interventions implemented. Nevertheless, since this was a cross-sectional sample, it may be regarded as representative of trained community pharmacists with varying experience with conducting CMRs.

## Conclusion

In the present study, conducted in daily practice, community pharmacists identified on mean three DRPs as the result of a CMR in older patients with polypharmacy. This number is similar to that of previous studies predominantly conducted in a research setting. Overtreatment and undertreatment accounted for less than half of the DRPs identified (41.4 %). More attention for drug classes regularly involved with DRPs may further decrease the number of DRPs. In dealing with DRPs, pharmacists proposed a variety of interventions of which the majority (69.9 %) was either implemented or led to alternative interventions. The application of explicit criteria (STOPP/START or the Amsterdam Tool) in a CMR may be useful in addressing and preventing DRPs frequently found in the medication of older patients with polypharmacy.
